# Competency Assessment in Training in African Countries

**DOI:** 10.3389/jaws.2025.14060

**Published:** 2025-08-29

**Authors:** Jacob A. Akoh

**Affiliations:** Nuffield Health Plymouth Hospital, Plymouth, United Kingdom

**Keywords:** mesh repair inguinal hernia, competency assessment, training, capacity building, feedback, index procedures

## Abstract

**Introduction:**

Progress through surgical training in many institutions in Africa is based on duration of apprenticeship, logbook activity, and success in prescribed examinations. Objective assessment of competency is less rigorous. This paper presents the outcome of two surgical training camps focused on open mesh repair of inguinal hernias and a comparison of trainee self-assessment with trainers’ assessment of their competency. It discusses the role of competency-based assessment in training of surgeons in Africa.

**Methods:**

A competency-based structured hernia training program lasting 8 days conducted in Uganda and Nigeria provided the materials for this study. Each day, a feedback session was held to discuss the trainee’s performance and learning. On the final day, the trainee’s performance was assessed using established criteria. The results were summarized using descriptive statistics and statistically analysed.

**Results:**

The training program resulted in a statistically significant rise in six specific parameters of knowledge and skill in hernia surgery (p < 0.0004). Of the 14 trainees, five were judged to be able to perform repair of small hernias independently and eight able to with minimal supervision or occasional help. With scrotal hernias, only one trainee was competent to perform repairs independently but 11 required occasional help only.

**Discussion:**

Training of junior surgeons or medical officers to a proficient level of competency that is safe and of good quality is possible within a few days as demonstrated by this and other reports. Feedback is critical to the success of competency-based training. Surgical Colleges in Africa need to select index procedures such as inguinal hernia repair that newly trained surgeons are required to be competent in to function.

**Conclusion:**

A shift to competency-based surgical training coupled with formative competency assessments in index procedures may lead to a more rapid manpower capacity building than the traditional approach to training of surgeons.

## Introduction

The surgical manpower crisis in Africa and much of the developing world is well known [1, 2]. Understandably, African countries have adopted several measures to address this crisis. Mozambique, Tanzania, Malawi, and Democratic Republic of the Congo started training non-physicians in surgical procedures, such as abscess drainage, hernia repair, and caesarean section. Niger has deployed a team of general practitioners certified as having “capacity in district surgery.” Senegal aims to train specialist surgeons and Rwanda has just announced a bold 4 × 4 program to train a quantum number of specialists in several fields [3, 4]. To guide the effort towards improving access to surgical care in low/middle income countries (LMIC), the Lancet Commission on Global Surgery determined the size of capacity building (2.2 million surgeons, anaesthetists, and obstetricians) and investment (350 billion USD) needed by 2030 to reach the Commissions’ goals [1].

Training a surgeon takes time and many training institutions in Africa have curricula that are time-limited with progress based on passing prescribed examinations. Because clinical governance is largely rudimentary in many parts of sub-Saharan Africa, it is difficult to know to what extent surgeons are held accountable for performing their tasks. Even more important is how one determines that a trainee surgeon has achieved competency in particular procedures or for a range of procedures. The pressure to recruit or train more surgeons may increase the temptation to overlook certain aspects/demands of training. A key feature of many training institutions in Africa is the use of logbooks to demonstrate how many cases the trainee has been involved in. This coupled with the duration of apprenticeship and the subjective opinions of senior surgeons are used to determine competency. Objective assessment of competency is therefore less rigorous, and decisions are often based on unsystematic observation with the attendant risks of bias and inconsistency. Traditionally surgeons justify their seniority or competency level on the number of years of practice and the sheer number of procedures performed.

The dogma that underlies the traditional approach to training is that expertise comes with experience. The questions to ask are how long it takes to learn by apprenticeship and how safe it is for patients if cared for by surgeons of unproven competency. Bladin et al. [5] conducted a register-based study to investigate the learning curve of surgeons performing open anterior mesh repair for groin hernias by assessing hernia recurrence rates, surgical complications, and operating times in relation to the number of procedures performed. They analysed 38,845 repairs performed by 663 resident general surgeons between 2005 and 2020 and concluded that 60 performed procedures during surgical residency is a reasonable target for achieving competency to perform groin hernia repairs safely without supervision. It is interesting that in their study, repairs by surgeons with fewer than 30 repairs were excluded. In a UK study, Abdelrahman and co-workers [6] examined the relationship between index operative experience and competency. They showed that the learning curve and caseload required to demonstrate level-4 consultant validated procedural-based assessment (L4C) related to specific procedure ranged from 0.76 to 3.4 times the national indicative target number guidance. Interestingly, they also showed that for inguinal hernias, the number to perform before achieving competency was 64 (17-132) cases. It is obvious that it would take too long to train surgeons if the key requirements for determining competency are the number of index procedures performed, and the duration of training. In a teaching hospital in Southwest Nigeria, one of two general surgery teams performed only 140 groin hernia repairs during a five-year period [unpublished presentation 2024]. Giving the large number of residents in the institution, it is difficult to determine how many of such residents would have the opportunity to achieve competency during their training if they had to repair 60 hernias each.

Competency assessment is about finding out if a person has the necessary skills and abilities to perform a task or set of tasks. Competency assessment is demanded by patients and the public because it is widely recognised as a vital component of ensuring adequate training of surgeons and giving good quality service to patients. Surgical training is transitioning from a time-based to a competency-based approach [7]. Many countries in sub-Saharan Africa, such as Zambia, Tanzania, South Africa, Rwanda, Nigeria, Kenya, and Ethiopia, have already undertaken ambitious competency-based education, while others are contemplating following suit [8]. A study from Cape town, South Africa shows that Africa is ready for a paradigm shift to competency-based assessment in surgical training [9].

This paper presents the outcome of a surgical training camp focused on open mesh repair of inguinal hernias and a comparison of trainee self-assessment with trainers’ assessment of their competency. It discusses the role of competency-based assessment in training of surgeons in Africa.

## Materials and Methods

A structured hernia training program held in Uganda in June 2024 and another in Nigeria in September 2024 provided the materials for this study. The programs were conducted along the general principles of previous camps organised by Operation Hernia (OH) [10]. The trainees received a one-day theoretical module on all pertinent aspects of abdominal wall hernias ranging from anatomy to tissue and mesh hernia repair under local/regional or general anaesthesia followed by hands-on training in theatre for 5 days. Information about the prior experience of the trainees was used to construct the different teams to ensure equitable distribution of experience. All efforts were concentrated on abdominal wall hernias during the week-long training with each doctor exposed to about 28–30 procedures (observing 10, assisting a senior surgeon in 10, performing assisted by a senior in 3 and by a colleague in 2-3 and assisting a colleague in 4–5). The trainee observed the trainer performing and demonstrating how to repair a hernia, assisted the senior surgeon and was allowed to perform aspects of the procedures, was assisted by the senior surgeon and eventually by another trainee with the senior surgeon observing. At the close of business each day, a feedback session was held to discuss the trainee’s performance and learning.

Eight trainees underwent intensive hands-on training under the guidance of three trainers during the Operation Hernia training camp in Uganda whereas six trainees had similar training in Nigeria with two trainers. The characteristics of the two training events are shown in Table 1. The purpose of the feedback and assessments was carefully explained to course participants. Apart from its use in the assessment process, it would be used to review the training in order to improve future courses. Information about the prior experience of trainees was obtained from questionnaires completed by the trainees. On the final day of camp, the trainee’s performance was assessed using the criteria set out in Tables 2, 3. As part of the competency-based training program, trainees were asked to complete questionnaires about aspects of their training. In addition to formative assessments of trainees during the week, a summative assessment of each trainee was made on the final day by their trainers. Trainees were asked to rate the knowledge and skills acquired on a scale (1 = poor, 10 = excellent). Both trainees and their trainers completed the competency questionnaires and the results presented in tabular form. For anonymity, the trainees were assigned numbers, known only to the author and relevant trainee. Hernias with sac contents not extending into the scrotum were regarded as simple hernias whereas those extending into the scrotum whether reducuible or not were regarded as scrotal or complex hernias.

**TABLE 1:** Comparison of two hernia training courses - Uganda and Nigeria.

Parameter	Uganda	Nigeria
Trainees	8	6
Trainers	3	2
Patients	62	51
Male	45 (72.6%)	47 (92.2%)
Female	17 (27.4%)	4 (7.8%)
Type of anaesthesia:		
General	5 (8.1%)	0
Spinal	28 (46.8%)	20 (39.2%)
Local	29 (45.1%)	31 (60.8%)
Procedures	77	56
Inguinal hernias	63	44
Umbilical/paraumbilical	3	5
Epigastric	7	1
Femoral	3	0
Spigelian	1	0
Hydroceles	0	6

**TABLE 2:** Specific parameters - trainees’ self-assessment of competence versus trainers’ (trainer scores in red) – UGANDA 2024.

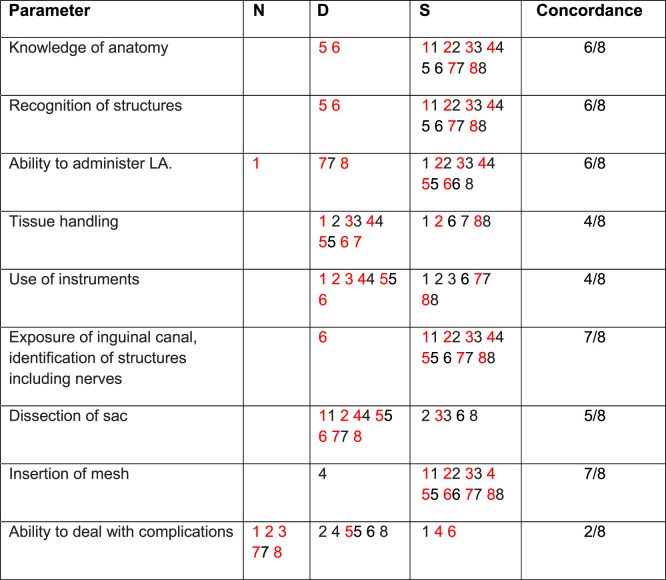

N: Not assessed; D: Development required; S: Satisfactory.

Trainees numbered 1-8.

**TABLE 3:** Specific parameters - trainees’ self-assessment of competence versus trainers’ (trainer scores in red) – NIGERIA 2024.

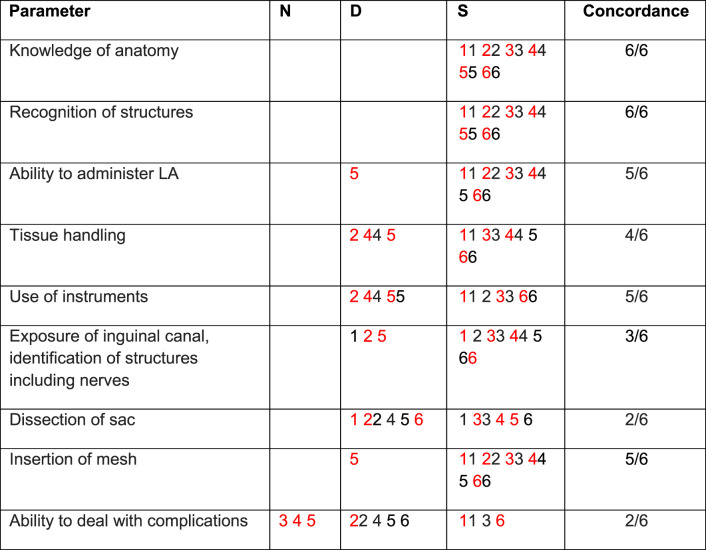

N, Not assessed; D, Development required; S, Satisfactory.

Trainees numbered 1-6.

### Analysis

Comparison of means of knowledge before and after the workshop was performed using the paired sample t-statistic. A value of P < 0.05 was deemed statistically significant.

## Results

**FIGURE 1:**
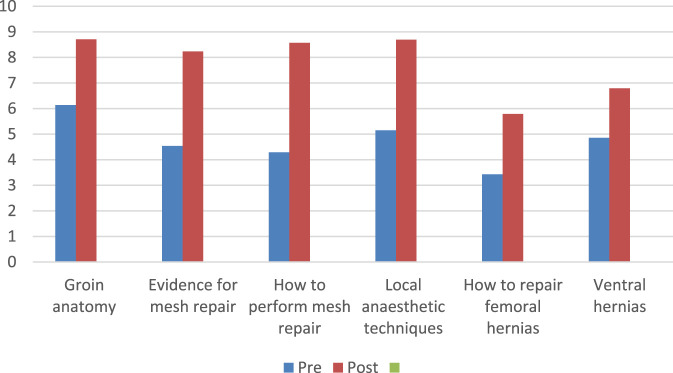
Self-assessment of pre- and post-course in six specific areas (all 14 trainees).

The training program resulted in a statistically significant rise in six specific parameters (p = 0.0004; difference in mean of pre- and post-course scores = −3.0617, 95% CI: -4.0146 to −2.1087) as shown in Figure 1. However, the difference between the mean scores (−2.9300) on self-assessment in overall knowledge and skill in hernia surgery before and after training was not quite statistically significant (p < 0.0607, 95% CI of difference: from −6.4877 to 0.6272).

Of the nine parameters used for assessment of competency (Tables 2, 3), concordance between trainee and trainer ranged from 2/9 to 8/9. All trainees were fully assessed by their trainers. However, two trainees did not rate their overall (global) competence on self-assessment. Of the remaining 12, there was concordance in 13 out of 24 scoring episodes with two trainees showing no concordance at all. With respect to anatomy (12/14) and recognition of structures (12/14), trainees and their trainers had a high level of agreement about trainees’ competency. However, with respect to ability to deal with complications (4/14), dissection of sac (7/14) and tissue handling (8/14) there was a low level of concordance with the trainees often rating themselves higher than their trainers’ rating.

Based on assessment by trainers, of the 14 trainees, five (36%) were judged to be able to perform repair of small hernias independently and eight (57%) able to with minimal supervision or occasional help leaving one trainee who required full supervision (Table 4). With scrotal hernias, only one trainee was competent to perform repairs independently but 11 required occasional help.

**TABLE 4:** Overall competency in mesh repair of inguinal hernia as determined by trainers.

Global judgement	Small hernias			Scrotal hernias		
	Ug	Nig	Total	Ug	Nig	Total
Unable to perform procedure						
Able to perform aspects of the procedure		1	1 (7.1%)	1	1	2 (14.3%)
Able to perform procedure with supervision	1	1	2 (14.3%)	2	2	4 (28.6%)
Able to perform with minimal supervision/occasional help	5	1	6 (42.9%)	4	3	7 (50.0%)
Able to perform independently	2	3	5 (35.7%)	1		1 (7.1%)
Total	8	6	14 (100%)	8	6	14 (100%)

Ug, Uganda; Nig, Nigeria.

Training in theatre as defined by five key components was rated as excellent by 55.7% and very good by 34.3% (Table 5). The sample size was too small to demonstrate any difference between the five teams.

**TABLE 5:** Trainees’ feedback on theatre training.

Topic	Average	Good	Very Good	Excellent
Demonstration of anatomy		1	2	11
Demonstration of various steps in hernia surgery		2	5	7
Demonstration of insertion of mesh		1	3	10
Hands on training in mesh repair	1		6	7
Your confidence in performing hernia repair	1	1	8	4
Total	2 (2.9%)	5 (7.1%)	24 (34.3%)	39 (55.7%)

## Discussion

Since 2016, UK and German charities have developed and employed a structured hernia surgical training program for postgraduate surgical trainees and medical doctors in Rwanda [10]. A similar program by UK surgeons has been ongoing in Uganda since 2016. The outcome of the 2024 surgical camps in Uganda and Nigeria where 13 of the 14 trainees (93%) were deemed able to perform small inguinal hernias either independently or with minimal supervision is better than the report by Lorenz and co-workers [10] stating that 20 of the 36 participants (55.55%) required only minimal supervision with four participants requiring full supervision after the completion of the course. Ashley and co-workers [11] have also demonstrated that short-course intensive hands-on training of medical doctors and associate clinicians in mesh hernia repair is effective and safe. Training of junior surgeons or medical officers to a proficient level of competency that is safe and of good quality is possible within a few days as demonstrated by both reports and other unpublished work in Uganda and Ghana [12]. Based on work done in Rwanda in 2023 and 2024 using a similar methodology, 22 of 47 general practitioners were able to perform simple inguinal hernias independently while only three were able to perform complex hernias independently [13]. The key features of these short-course training programs are concentrated training coupled with assessment of competency using established criteria.

The features of competency-based assessment include defining the criteria to be used, evidence required, type of binary judgement, level of participation by trainees, and choice of assessment pathway. Use of self-assessment questionnaires, 360-degree feedback surveys, skills tests, interviews, observations, and simulations may increase the robustness and validity of the process. New technologies such as objective structured assessment of technical skills (OSATS) [14]; hand motion analysis–Imperial College Surgical Assessment Device (ICSAD) [15]; ADEPT–Advanced Dundee Endoscopic Psychomotor Tester [16] and virtual reality [16] though not readily available in Africa, may facilitate objective assessment of technical skill. Simulation is increasingly being explored as an assessment modality. Using a simulation-based model designed to assess the operative competence of higher specialist trainees in general surgery, Toale et al [17]. demonstrated a significant difference in mean station score between junior and senior trainees. A study conducted by Traynor and co-workers [18] in 2021 showed that simulation is a valuable educational tool that should be included in training in Africa. Also, using a smart phone based operative evaluation application (SIMPL), residents can be evaluated performing common surgical procedures [7]. Our post training camp mentoring program is considering adopting a system like SIMPL to provide ongoing assessment and help to trainees who did not attain independent status.

To realise the full benefits of competency assessment in surgical training, competency based medical education and training must be instituted. This requires careful planning and reshaping of the medical curricula [19]. This must promote learner-centredness; de-emphasise time-based training; explicitly define competency and its subcomponents; select educational activities, experiences, and instructional methods; and finally select assessment tools to measure progress. The training must define the range of knowledge, skill, and understanding that an individual should have achieved at a certain stage of their career. It must also be accompanied by carefully designed tasks that appropriately and accurately sample and estimate level of competency with cut-off points to separate the competent from the not yet or barely competent person.

The competency assessment tool chosen must gain the confidence of regulatory bodies. For example, the use of entrustable professional activities (EPAs) was endorsed by the American Board of Surgery following a 2-year feasibility pilot study [20]. Confidence in EPA summative entrustment decisions increased as the number of EPA assessments increased, providing early validity evidence for this novel assessment framework. The key seems to be defining what the index EPAs should be. Combining surgical videos and structured assessments may increase the reliability and validity of independent competency evaluations. Nikolian et al. [21] used a novel objective, procedure-specific, 8-item competency assessment for minimally invasive inguinal hernia repair to test inter-rater reliability using a “safe” vs. “unsafe” scoring rubric and felt this may work well with the American Board of Surgery’s EPAs initiative. Simulation platforms, whether deployed physically or online may be used to improve OSCE type examinations. Liebert and co-workers [22] showed on multivariate analysis, there was a strong association between MCS (Membership of the College of Surgeons) percentage score and ENTRUST (online virtual patient simulation platform) total score (p < 0.001). ENTRUST may be a useful learning and assessment platform for surgical trainees as it appears to discriminate the competent from non-competent candidates.

Even without the newer methods, assessment of technical performance is a strong component of the Operation Hernia training program. Clinical judgement is assessed in terms of trainee’s diagnostic ability and formulation of the treatment plan–this is done during the pre-theatre visit on the ward. These two factors influence the decision-making process during a surgical procedure. The theoretical workshop prior to the theatre training session enhances clinical judgement and provides the knowledge base required to implement decisions made while the direct observation of the psychomotor aspects of tasks at hand makes for a comprehensive assessment of this training.

Feedback is critical to the success of competency-based training and must feature prominently in assessment of competency. To be valuable, feedback must be contemporaneous, confidential, security conscious, constructive, evidenced, developmental and followed by SMART (specific, measurable, achievable, relevant, and time-bound) actions. Given the volume of cases available during the training camp, trainees had the opportunity to address short comings almost immediately. The findings in this study suggest that concordance between trainees and trainers is low and highlights the importance of feedback in the assessment process. As reported by Lorenz and co-workers [13], self-assessment by trainees tend to be more positive than those of their trainers. This is particular so in areas such as instrument and tissue handling when experience and judgement are required to make a decision.

Institutions in advanced countries already use a selection of index procedures to aid their competency-assessment in training [6, 23]. Using the Supervised Structured Assessment of Operative Performance (SSAOP) tool, the operative competence of core surgical trainees (CSTs) in Ireland, were analysed across a mix of undifferentiated procedures, as well as for three commonly performed general surgery procedures: appendicectomy, abdominal wall hernia repair, and skin/subcutaneous lesion excision. Following analysis of 2,294 SSAOPs, they concluded that trainers should conduct repeated assessments across a smaller number of index procedures to improve reliability [23]. Countries and Surgical Colleges in Africa need to select from the following inexhaustive list, the procedures that newly trained surgeons are required to be competent in to function well and improve access to surgical care. Such index procedures may include emergency laparotomy, appendicectomy, mesh repair of inguinal hernia, Hartmann resection, segmental colectomy, laparoscopic cholecystectomy, laparotomy for trauma, incisional hernia, mastectomy, and thyroidectomy. The choice of inguinal hernia repair is because hernias are common (about 15% of general surgical procedures) and the skills acquired in hernia training are both generic and transferrable. Furthermore, the global relevance of hernia surgery as a tracer conditionfoe evaluating access to and quality of elective care as reported by Lancet Global Health [24] undescores the importance of improving hernia surgery outcomes by effective training such as reported in this study.

Competency assessment in surgical training is aimed at producing surgeons capable of handling carefully defined challenges. Such an approach has the advantage of shortening the duration of training and providing the much-needed surgical manpower to improve access to surgical care in the community. The surgeons would have the opportunity to continue their professional development on the job. Structured training in hernia surgery equips trainees with generic and transferable skills to other conditions.

This study has a few limitations. The small number of trainees coupled with the fact that not all participants completed the questionnaires correctly or consistently restricts the ability to generalise the outcomes of the study. Also, a degree of subjectivity is unavoidable with the various assessments. Nevertheless, trainees have benefited immensely judging by their feedback and experience after the camp. Moreover, the results of these training camps in two African countries were similar. This provides strong support for extending this type of competency based training to other countries in Africa. A similar outcome from a larger cohort of 47 general practitioners in Rwanda [13] lends support to the reproducibility of the findings of this study. Another limitation of this study is the absence of a formalised follow up to assess skill retention. However, the reports were made accessible to trainees, local trainers and mentors to enable long term mentoring and the creation of opportunities for trainees to continue to sharpen their skills. A future study will explore skill retention and experience of trainees post structured hernia training.

## Conclusion

This report demonstrates that, medical personnel in Africa can be trained in hernia repair with mesh using a short duration structured training programme. With further refinement of the training program including simulation and post training mentoring, and focused training in other key areas, competency based assessment may be upscaled and made more beneficial to the training of surgeons in Africa. The College of Surgeons of East, Central and South Africa and other postgraduate medical or surgical colleges should determine what index procedures are required for training surgeons suitable for meeting the basic needs of African countries. Such key procedures, which must include inguinal hernia repair, must be accompanied by clear objectives about the level of competency surgeons must attain. Based on experience with inguinal hernias, a shift to competency based surgical training coupled with formative competency assessments may lead to a more rapid manpower capacity building than the traditional approach to training of surgeons. Charitable organisations such as Operation Hernia and overseas Surgical Colleges working in collaboration with African Surgical Colleges may ensure faster progress in this drive. The road to surgical independence in Africa lies in mastery of small challenges. The time is ripe for competency-based training as the best way to produce safe and competent surgeons that are much needed in Africa.

## Data Availability

The raw data supporting the conclusions of this article will be made available by the authors, without undue reservation.
